# Efficacy and safety of topical spironolactone 5% cream in the treatment of acne: A pilot study

**DOI:** 10.1002/hsr2.317

**Published:** 2021-07-01

**Authors:** Azin Ayatollahi, Ansieh Samadi, Ayda Bahmanjahromi, Reza M. Robati

**Affiliations:** ^1^ Center for Research and Training in Skin Diseases and Leprosy Tehran University of Medical Sciences Tehran Iran; ^2^ Skin Research Center Shahid Beheshti University of Medical Sciences Tehran Iran; ^3^ Department of Dermatology Loghman Hakim Hospital, Shahid Beheshti University of Medical Sciences Tehran Iran

**Keywords:** acne, anti‐androgen, spironolactone, topical, treatment

## Abstract

**Background:**

Spironolactone is an effective treatment for female patients with acne vulgaris. However, topical spironolactone could be a valuable treatment option in both male and female acne patients due to the less possibility of systemic side effects with its topical formulation.

**Objective:**

To evaluate the efficacy and safety of 5% spironolactone cream in the treatment of mild to moderate acne vulgaris.

**Methods:**

In this pilot clinical trial, topical spironolactone 5% was evaluated to treat patients with mild to moderate acne twice a day for 8 weeks. The rate of improvement as any alterations in the number of open and closed comedones, facial inflammatory papules, and acne global grading scores were assessed. Moreover, skin biometric characteristics including skin hydration, erythema, transepidermal water loss (TEWL), pH, sebum, and *Propionibacterium acnes* bacteria activity were also assessed following the treatment.

**Results:**

Fifteen patients participated in our study with a mean age of 25 ± 4.87 years old. A total of 66.6% (*n* = 10) were female and 33.4% (*n* = 5) were male. The number of acne papules, open and closed comedones, and acne global grading score decreased significantly 4 and 8 weeks after the beginning of treatment (*P* < .05). No considerable side effect was reported. Moreover, there was no significant difference between the skin hydration, melanin, erythema, TEWL, pH index, sebum, and *P acnes* bacteria activity before, 4, and 8 weeks after the treatment with topical spironolactone cream (*P* > .05).

**Conclusion:**

The topical 5% spironolactone cream seems to be an effective and safe treatment of acne vulgaris in both male and female patients.

## 1 INTRODUCTION

Acne is a chronic inflammatory disease of the pilosebaceous unit resulting from androgen‐induced increased sebum production, altered keratinization, inflammation, and bacterial colonization of hair follicles by *Propionibacterium acnes*. It affects millions of people worldwide and persists into adulthood in approximately 12% to 14% of cases with undeniable psychological and social consequences.^1^, ^2^, ^3^ Proper evaluation of the type and severity of acne is essential for successful management. Mild acne can be treated by topical treatment only, whereas moderate and severe acne need systemic treatment in addition to topical treatment.^4^

Spironolactone (SP) is an aldosterone antagonist with anti‐androgen properties. SP's mechanism of action includes blocking 5α‐reductase activity through increasing testosterone clearance as a result of enhanced liver hydroxylase activity.^5^ Reported side effects include breast tenderness, urinary frequency, menstrual irregularities, hyperkalemia, fatigue, headache, dizziness, lethargy, hypotension, and birth defects.^6^, ^7^

However, the use of a topical form of spironolactone could have similar efficacy with lower possible side effects. There are few available studies about the efficacy of topical spironolactone in the treatment of acne. In this study, we plan to evaluate the efficacy and safety of 5% topical spironolactone cream in the treatment of acne vulgaris.

## 2 PATIENTS AND METHODS

This pilot study was designed and performed on patients with mild to moderate facial acne based on the provided inclusion and exclusion criteria. These patients were referred to the dermatology clinic of our center from May 2017 to April 2018. The study was approved by the research ethics committee of Tehran University of Medical Sciences (IR.TUMS.VCR.REC.1396.2010) and was performed under the Helsinki research ethics statement. All the participants also signed the project informed consent form.

Criteria for subjects included in the study were age between 18 and 40 years old, mild to moderate acne, lack of change in the diet, and lifestyle during study time. The exclusion criteria were the history of taking the systemic anti‐acne treatment during the last 4 weeks, the use of topical anti‐acne medications in the last 2 weeks, the use of isotretinoin, laser therapy, and peeling in the last 6 months, pregnancy, and lactation. The spironolactone 5% cream, prepared by a pharmacist, was given to the patient for topical use at the beginning of the treatment. Instructions for using the cream twice a day (morning and evening) on the face were discussed for the patients. They were also instructed to first wash their skin with baby soap and water, and after drying the skin, rubbed a certain amount of cream on the face, especially in areas with acne lesions, and after 2 hours, washed their face with water. The duration of the study was 8 weeks and the patient visited at the beginning of the study, 4, and 8 weeks after the start of treatment. A questionnaire form containing the person's demographic information, type and severity of acne, the duration of the acne lesions, history of previous acne treatments, and possible side effects was provided. Before the study began, in the fourth and eighth weeks after initiation, photographs were taken using a specific camera with the same light and background in all cases. Acne grading was conducted through the Acne Global Grading System.^8^ In each of the visits, the patient was also asked about complications such as erythema, itching, burning, etc.

The activity of the propionibacterium acne bacteria was assessed with Visiopor PP 34 N before treatment, 4, and 8 weeks after treatment for porphyrin (bacterial pigmentation) observation. The camera used ultraviolet light to detect the corresponding fluorescence in acne lesions in an area of 10 × 8 mm. The number of facial acne lesions including inflammatory papules, open and closed comedones was also counted.

Skin sebum, pH, transepidermal water loss (TEWL), hydration, and erythema (along with melanin) were also evaluated by Sebumeter SM 815, pHmeter PH 905, TEWA meter (TM 300), Corneometer CM 825, and Mexameter MX 18 MPA (Courage and Khazaka electronic GmbH, Cologne, Germany), respectively, before and 4 to 8 weeks after treatment. These measurements were conducted at room temperature 24°C to 26°C with a relative humidity of 50% ± 3%.

Statistical analyses were conducted using SPSS 16.0.0 software. *P*‐value of less than .05 was considered statistically significant. To collect demographic information, mean and SD, and relative frequency were used. According to the research objectives, the *t* test was used for data analysis.

## 3 RESULTS

Fifteen patients participated in our study. A total of 66.6% (*n* = 10) were female participants and 33.4% (*n* = 5) were male. The mean age of the participants in the study was 25 ± 4.87. In addition, the oldest in our study was 35 years and the youngest was 18 years old. Of these 15 patients, 3 subjects did not come for follow‐up, and two subjects came just one time for follow‐up.

**FIGURE 1:**
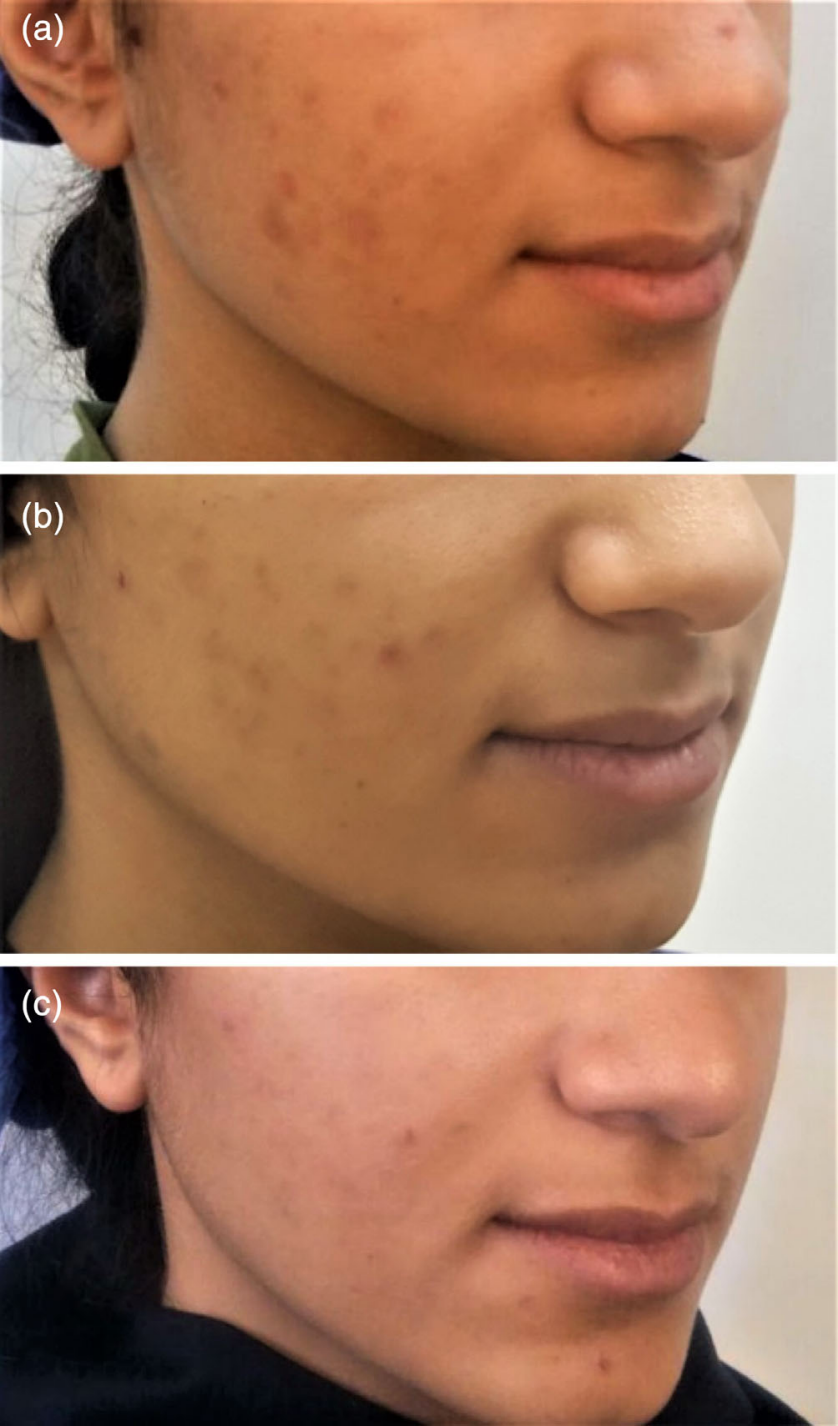
Moderate improvement of acne lesions with topical spironolactone 5% cream (A, before treatment, B, 4 weeks, and C, 8 weeks after treatment)

The mean number of papules were 9.00 ± 4.94, 2.98 ± 3.12, 2.37 ± 1.76, before treatment with topical spironolactone, after 4 and 8 weeks of treatment, respectively. There was a significant decrease in the mean number of papules after 4 weeks (*P* = .000), and 8 weeks of treatment (*P* = .004) (Table 1). The mean number of open comedones in the patients before, after 4, and 8 weeks of treatment were 2.6 ± 1.07, 1.7 ± 1.06, and 0.75 ± 0.88, respectively. There was also a significant decrease of open comedones 4 and 8 weeks after treatment (*P* = .000) (Table 1). The mean number of closed comedones before, after 4, and 8 weeks of treatment were 20.3 ± 6.88, 14.1 ± 4.65, and 9.25 ± 4.06, respectively. Our results show that there was a significant decrease in the number of closed comedones 4 and 8 weeks after treatment with topical spironolactone (*P* < .05). Finally, we examined the acne grading score in patients and our results showed that the mean grading score in patients before, after 4, and 8 weeks of treatment were 3.75 ± 0.70, 1.9 ± 0.56, and 1.37 ± 0.51, respectively. There was a significant decrease in the grading score of patients before and 4 and 8 weeks after treatment with topical spironolactone (*P* < .05) (Table 1 and Figure 1).

**TABLE 1:** Study variables characteristics before and after treatment with topical spironolactone

Variable	Before administration	Four weeks after administration	Eight weeks after administration	p1^a^	p2^b^
Mean number of facial acne lesions	31.4 ± 16.24	14.9 ± 11.97	14.50 ± 12.18	0.15	0.034
Inflammatory papules (mean number)	9.0 ± 4.94	2.98 ± 3.12	2.37 ± 1.76	0.000	0.004
Open comedones (mean number)	2.6 ± 1.07	1.7 ± 1.06	0.75 ± .88	0.010	0.000
Closed comedones (mean number)	20.30 ± 14.10	4.65 ± 4.65	9.25 ± 4.06	0.000	0.000
Mean acne global grading scale	3.75 ± 0.70	1.9 ± .56	1.37 ± 0.057	0.000	0.033
Mean *P. acne* bacteria activity	1.44 ± 0.92	1.47 ± 1.32	1.07 ± 0.97	0.944	0.597
Mean skin hydration index	56.65 ± 13.457	57.85 ± 12.82	52.51 ± 19.35	0.749	0.567
Mean melanin index	201.26 ± 43.69	205.31 ± 39.3	218.00 ± 38.502	0.310	0.111

^a^ p1: Comparing before the study and 4 weeks after study.

^b^ p2: Comparing before the study and 8 weeks after study.

**FIGURE 2:**
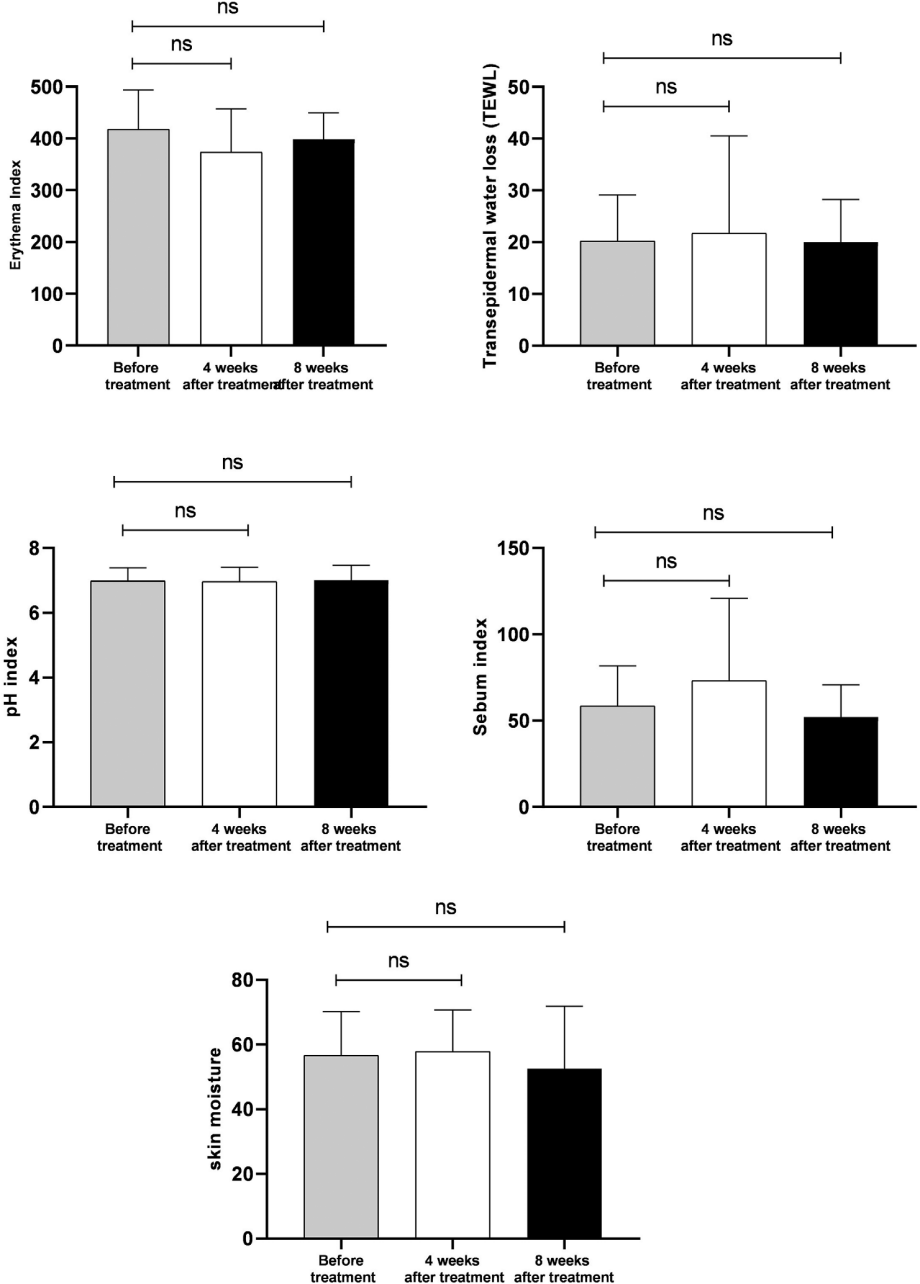
Skin biophysical characteristic before, 4 and 8 weeks after treatment (ns, not significant)

Patients did not show any side effects at each follow‐up. Moreover, there was no significant difference between the skin hydration index, melanin index, erythema, TEWL, pH index, sebum index, and *P acnes* bacteria activity before, 4, and 8 weeks after the treatment with topical spironolactone 5% cream (*P* > .05) (Table 1 and Figure 2).

## 4 DISCUSSION

Topical Spironolactone (SP) known as an anti‐androgen drug, has been proven to be effective in the treatment of acne. Recently, there is growing interest to prepare SP in different topical formulations to minimize the unnecessary systemic side effects associated with the oral drug administration of spironolactone.^9^, ^10^

In our study, we used topical SP 5% cream as a treatment of acne in 8 weeks. This topical treatment shows significant improvement of acne as the significant decrease in the mean number of acne papules, open and closed comedones. The acne grading scores were also showed significant improvement with topical SP 5% cream. Moreover, our patients did not experience any severe side effects. The topical SP 5% cream seems not to cause any systemic endocrine side effects even in men and its use could be safe in male patients with acne.^11^ Therefore, it might be a good topical treatment option in both male and female patients with acne.

We also found that topical spironolactone 5% cream did not significantly change skin redness, skin sebum, skin moisture, and pH levels among the patients. These findings could also imply the safety of topical SP 5% cream, which does not cause skin dryness, erythema, and sensitivity. The amount of sebum measured by the Sebumeter did not make any significant difference following the treatment with topical SP 5% cream. However, this device only measures the sebum on the surface of the skin. Therefore, it is not possible to check the activity of sebaceous glands completely and further studies are needed in this regard. Moreover, there are some controversies about the effect of topical SP on sebum secretion. Some studies indicate the reduction of sebum secretion but others show no significant effect of this drug on skin sebum content.^12^, ^13^

There are limited studies in the literature that evaluate topical spironolactone formulations to treat acne vulgaris with considerable efficacy. In some of these studies, topical spironolactone 5% gel has been used^14^, ^15^ but others use this drug in cream formulation similar to our study.^16^ Because the gel base contains ethanol, it may irritate the skin and make the patient feel skin dryness due to the evaporation of water from the gel base. The reduction of skin sebum content in these studies may also be due to the effect of ethanol in the gel‐based formulation.^14^

Our study revealed that the number and severity of acne lesions decreased significantly over 8 weeks with topical spironolactone 5% cream, which can be attributed to its rapid and continuous effect. There was also no change in skin biophysical characteristics including skin erythema, TEWL, and pH, which could imply that this formulation showing no serious side effects. The main limitations of our study were its small sample size, lack of a control group, and the short period of follow‐up. Moreover, we could not assess any hormonal parameters before and after treatment to assess the possible systemic absorption of topical SP due to our limited facilities. However, it seems to be a distinctive study that evaluates the efficacy and safety of spironolactone cream both clinically and also objectively by skin biometric assessment. Therefore, future controlled studies, with more participants along with serum hormonal assessments and skin biophysical evaluations would be beneficial to elucidate and confirm the definite efficacy and safety of topical spironolactone cream both clinically and also in the aspect of skin biometric alterations or any systemic absorption of topical SP.

## CONFLICT OF INTEREST

The authors declare no conflicts of interest.

## AUTHOR CONTRIBUTIONS

Conceptualization: Azin Ayatollahi

Data curation: Ansieh Samadi

Investigation: Azin Ayatollahi, Reza M. Robati

Methodology: Azin Ayatollahi, Ansieh Samadi

Project administration: Azin Ayatollahi

Resources: Azin Ayatollahi

Supervision: Azin Ayatollahi, Reza M. Robati

Writing ‐ original draft preparation: Azin Ayatollahi, Ansieh Samadi, Ayda Bahmanjahromi, Reza M. Robati

Writing ‐ review and editing: Azin Ayatollahi, Ayda Bahmanjahromi, Reza M. Robati

Azin Ayatollahi and Reza M Robati confirm that they had full access to all of the data in the study and take complete responsibility for the integrity of the data and the accuracy of the data analysis.

## TRANSPARENCY STATEMENT

All the authors affirm that this manuscript is an honest, accurate, and transparent account of the study being reported, and no important aspects of the study have been omitted.

## ETHICS STATEMENT

The study was approved by the research ethics committee of Tehran University of Medical Sciences (IR.TUMS.VCR.REC.1396.2010) and was performed under the Helsinki research ethics statement. All the participants signed the project informed consent form.

## Data Availability

Related data of this project are available on request.
